# A Case of Intrauterine Listeria Infection During Pregnancy: NanoSuit Imaging of Listeria monocytogenes in the Amniotic Membrane

**DOI:** 10.7759/cureus.87792

**Published:** 2025-07-12

**Authors:** Chihiro Dohshita, Naomi Isomura, Chizuko Yaguchi, Akira Ohishi, Hiroaki Itoh, Tomomi Kotani, Hideya Kawasaki

**Affiliations:** 1 Department of Obstetrics and Gynecology, Hamamatsu University School of Medicine, Hamamatsu, JPN; 2 Department of Pediatrics/Maternal-Fetal Neonatal Care Center, Hamamatsu University School of Medicine, Hamamatsu, JPN; 3 Department of Pediatrics, Hamamatsu University School of Medicine, Hamamatsu, JPN; 4 Department of Obstetrics and Gynecology, Fujieda Municipal General Hospital, Fujieda, JPN; 5 NanoSuit Research Laboratory, Division of Preeminent Bioimaging Research, Institute of Photonics Medicine, Hamamatsu University School of Medicine, Hamamatsu, JPN

**Keywords:** intrauterine infection, listeria monocytogenes, nanosuit imaging, pregnancy, preterm delivery

## Abstract

Listeriosis during pregnancy is rare but can lead to premature miscarriage and intrauterine fetal death. A 29-year-old pregnant woman at 29 weeks of gestation was transferred to our hospital because of mild fever and abdominal pain. Emergency cesarean section was performed due to non-reassuring fetal status concomitant with suspected intrauterine infection. An unusually bright yellow amniotic fluid was observed. The oropharyngeal cavity of the neonate was occupied by thick, tenacious yellow mucus, making its removal challenging. The neonate was intubated due to poor oxygenation. Neonatal blood cultures revealed *Listeria monocytogenes*. Gram staining of cerebrospinal fluid was negative. The neonate was treated and discharged on the 65th day after birth. The mother had a fever of 39.4°C on the first day after surgery; however, no other significant incident occurred. She was discharged on the 10th day after surgery. Placental pathology revealed funisitis, chorioamnionitis, and marginal deciduitis without evidence of villitis, suggesting predominant transvaginal rather than hematogenous infection. Scanning electron microscopy using NanoSuit imaging revealed *Listeria monocytogenes* in the amniotic epithelium. The presence of *Listeria monocytogenes* has been reported in foods stored in refrigerators for extended periods of time and in ready-to-eat meals; therefore, it is difficult for pregnant women to be aware of all potential risks. It is important for physicians to recognize that listeriosis may have a long incubation period and present with mild maternal symptoms. Nevertheless, it should be considered as a differential diagnosis. Routine and prompt identification of the causative organism through amniotic fluid and placental swab cultures is important, particularly when intrauterine infection is suspected. Moreover, pathological examination of the placenta can provide insights into the potential route of infection.

## Introduction

Listeriosis is a zoonosis transmitted by ingestion of foods containing *Listeria monocytogenes*, a facultative anaerobic Gram-positive rod bacterium. Although listeriosis is rare, its incidence is approximately 13 times higher in pregnant women than in non-pregnant individuals [1]. Iida et al. reported that the number of reported cases of listeriosis has only been about 30 sporadic cases per year in Japan, which has a population of 120 million [2]. Furthermore, Okutani et al. estimated the annual incidence of listeriosis in Japan as 0.65 cases per one million inhabitants [3]. The incubation period can range from a few days to three months, and even mild maternal symptoms may lead to serious outcomes such as fetal growth restriction, fetal dysfunction, or intrauterine fetal death [4]. The present study included NanoSuit-enhanced scanning electron microscopy imaging that identified *Listeria monocytogenes* in the amniotic membrane epithelium in the case of chorioamnionitis. Herein, we propose a route of transvaginal infection through placental pathology.

## Case presentation

A 29-year-old woman, gravida 1 para 0, had a slight fever of 37.7°C and abdominal pain at night at 29 weeks of gestation. She visited her primary care provider the next day. Uterine contractions were observed every two to three minutes, there was bloody vaginal discharge, and the cervical length had shortened to 15 mm. Blood tests revealed inflammatory responses; however, influenza and severe acute respiratory syndrome coronavirus 2 (Sars-CoV-2) antigen tests were negative. She was transported to our hospital. On admission, she was awake and alert, with a body temperature of 37.3°C, pulse rate of 89 beats/min, blood pressure of 124/63 mmHg, respiratory rate of 20 breaths/min, and oxygen saturation of 96% (on room air). She had periodic uterine contractions and lower abdominal pain with a moderate amount of bloody vaginal discharge. Transabdominal ultrasound showed no placental thickening, with the fetus in breech presentation. The estimated fetal weight was -0.4 standard deviations, and the amniotic fluid volume was within the normal range. Blood tests revealed inflammatory responses (Table 1).

**Table 1:** Laboratory findings on admission BUN: Blood Urea Nitrogen, LDH: Lactate Dehydrogenase, ALT: Alanine Aminotransferase, AST: Aspartate Aminotransferase, ALP: Alkaline Phosphatase, γ-GTP: γ-Glutamyl Transpeptidase.

Test parameter	Value	Reference range
Hemoglobin	10.7	11.6－14.8g/dL
White Blood cells	19610	3300－8600/μL
Platelets	30.2×10⁴	15.8－34.8×10⁴/μL
C-reactive protein	9.48	0－0.1mg/dL
Creatinin	0.55	0.46－0.7mg/dL
BUN	4.7	8.0－20.0mg/dL
Total bilirubin	1.23	0.4－1.5mg/dL
Total protein	6.2	6.6－8.1g/dL
Albumin	3	4.1－5.1g/dL
A:G retio	0.94	1.32－2.23
LDH	145	124－222U/L
ALT	41	7－23U/L
AST	21	13－30U/L
ALP	133	38-113U/L
γ-GTP	24	9-32U/L
PT	13.4	10.0－13.0SEC
APTT	35.1	24.0－36.0SEC
PT-INR	1.04	0.80ー1.20SEC

In consideration of the maternal signs of infection and indeterminate group B streptococcus status, ampicillin was administered as the initial empirical antibiotic. An emergency cesarean section was performed based on a diagnosis of non-reassuring fetal status from fetal heart rate monitoring, concomitant with a possible intrauterine infection. During the operation, an unusually bright, clear lemon color amniotic fluid was observed. After delivery, oxytocin and methylergometrine were administered for poor uterine contractions. Blood loss was 2,400 g. After surgery, the antibiotic was changed to cefazolin sodium in accordance with the perioperative antibiotic guidelines in the hospital. On the first day after surgery, hemoglobin (Hb) was 7.2 g/dL. We administered ferric carboxymaltose 500 mg. Skin and blood cultures from the neonate, as well as placental scraping cultures, were positive for *Listeria monocytogenes*. As a result, the antibiotic regimen was switched to sulbactam/ampicillin. The mother had a fever of 39.4°C on the first day after surgery; however, no other significant incident occurred. After changing to oral antibiotics, she was discharged on the 10th day after surgery. Maternal blood, fecal, urine, and vaginal cultures were all negative.

**Figure 1:**
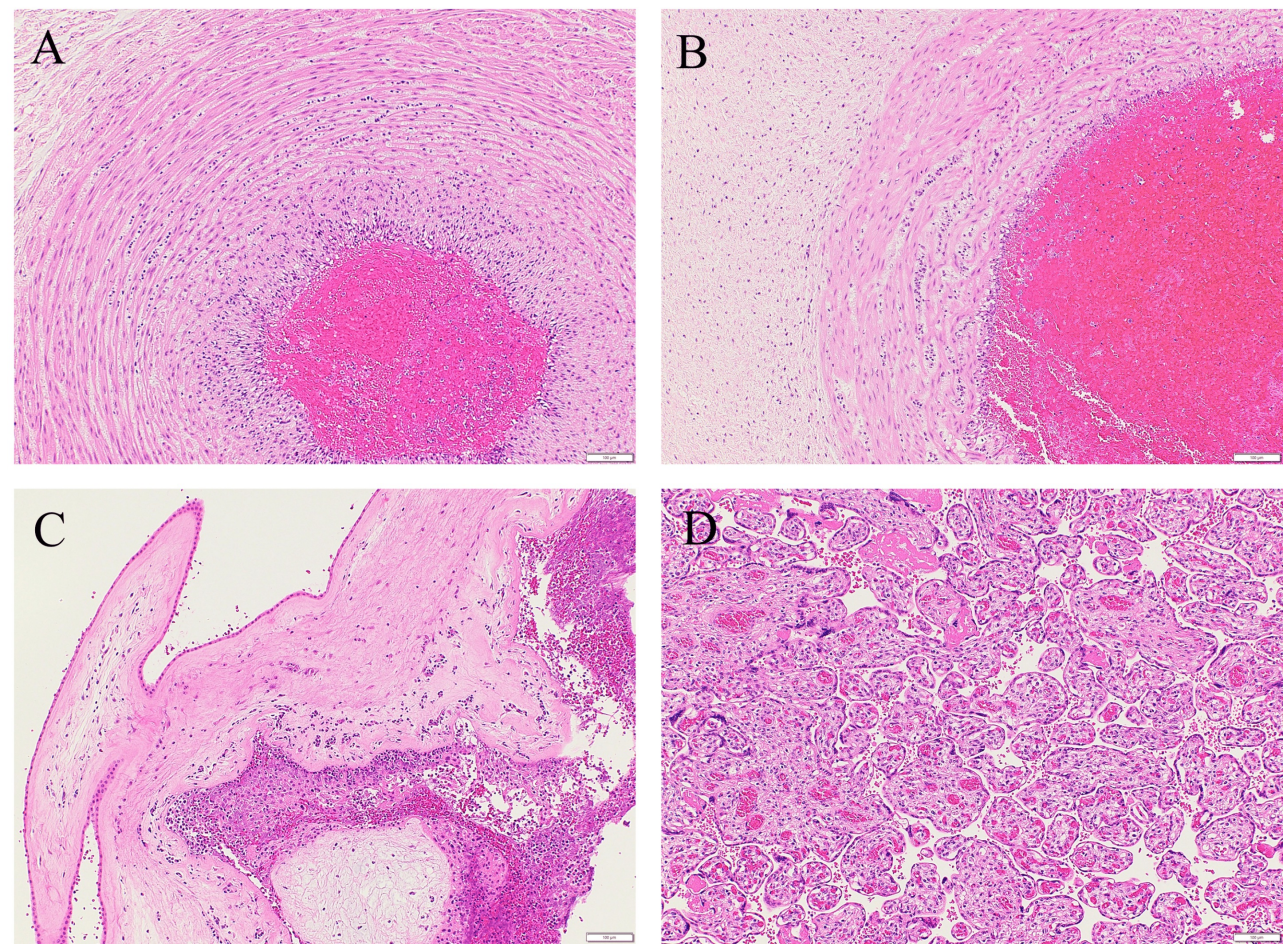
Placental pathological findings (H&E staining: scale bar 100μm) A: Umbilical artery - neutrophil infiltration extending into the muscular layer of the vascular wall, B: Umbilical vein - neutrophil infiltration extending to Wharton’s jelly, C: The infiltration of numerous neutrophils is observed on the decidual side of the marginal placental membrane, D: Neutrophil infiltration is not observed in the placental villi.

The newborn weighed 1366 g (0.0 standard deviations), had an Apgar score of 1/1 (one minute/five minutes), and an umbilical artery blood pH of 7.331. The oropharyngeal cavity of the neonate was occupied by thick, tenacious yellow mucus, making its removal challenging. Artificial respiration was initiated due to respiratory distress, and intubation was required because of bradycardia. Gradually, the patient’s heart rate increased, and SpO2 reached 100%. Mechanical ventilation with inhaled nitric oxide was started for pulmonary hypertension. Platelet count was 60,000/μl, and blood transfusion was initiated. Polyethylene glycol-treated human immunoglobulin was administered, and empirical antibiotic therapy was initiated with Ampicillin and cefotaxime. On the second day, the newborn was extubated. Blood cultures became negative on the second day after birth. Gram staining of cerebrospinal fluid was negative. The neonate was discharged on the 65th day after birth. Pathological examination by H&E staining showed massive neutrophil infiltration around the umbilical vessels, extending to Wharton’s jelly (Figures 1A, 1B), chorioamnion, and the decidual side of the marginal placental membrane(Figure 1C), but not in the villi (Figure 1D).

**Figure 2:**
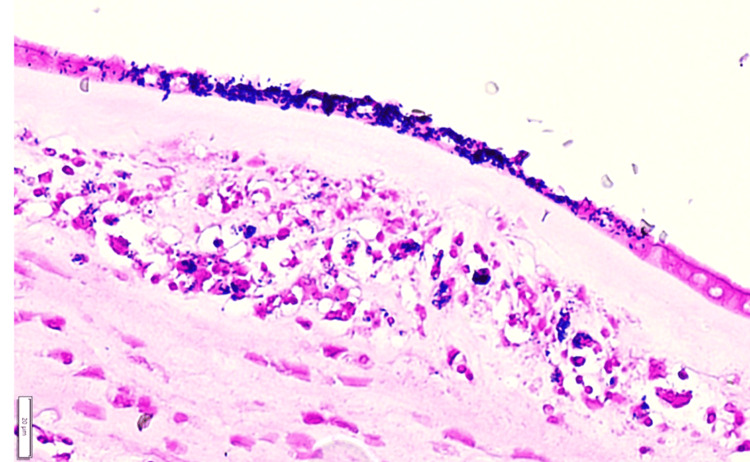
Amniotic epithelium was found to be Gram stain positive Gram stain findings (scale bar; 20μm).

Gram stain findings were positive in the amniotic epithelium (Figure 2).

**Figure 3:**
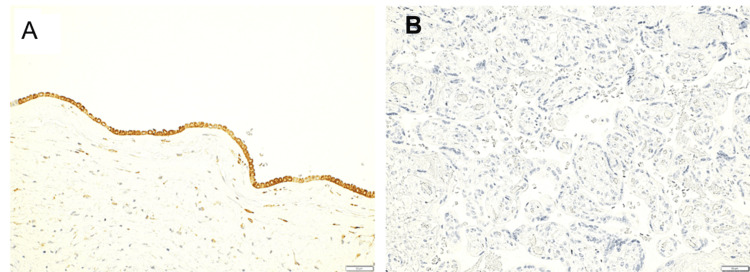
Immunohistochemical staining of Listeria monocytogenes Anti-Listeria antibody: abcum:ab35132 (scale bar; 50μm). A: Positive (brown) is observed in the amniotic epithelium, B: Positive staining of Listeria monocytogenes is not observed in the placental villi or intervillous spaces.

Immunohistochemical staining for *Listeria monocytogenes* showed positive staining in the amniotic epithelium (Figure 3A), but not in the villi (Figure 3B).

**Figure 4:**
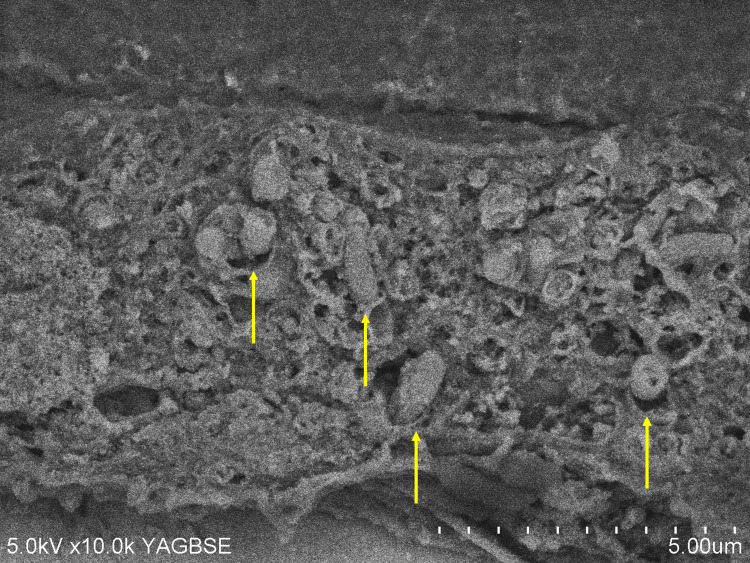
NanoSuit-assisted scanning electron microscopy reveals Listeria monocytogenes in the amniotic membrane epithelium Numerous bodies of *Listeria monocytogenes* (yellow arrows) are observed in the amniotic epithelium.

Moreover, scanning electron microscopy with NanoSuit, performed according to the methodology of a previous study [5,6], revealed numerous *Listeria monocytogenes* within the amniotic epithelium (Figure 4).

The NanoSuit method enabled live observation of fly larvae under a high-vacuum scanning electron microscope (SEM). By simply applying the NanoSuit solution, the tissue retains its hydration and original structure, allowing for clear SEM imaging without the need for traditional fixation or dehydration. Performed immunohistochemical staining for Listeria on formalin-fixed, paraffin-embedded sections. Captured images of the 3,3'-diaminobenzidine (DAB)-positive Listeria-stained areas under a light microscope, and marked these areas on both the front and back of the slide using a solvent-resistant pen. By incubating the slide glass, after immunostaining, in a 1% osmium solution for five minutes, the osmium will bind specifically to DAB, enhancing DAB-positive regions for SEM observation. After rinsing with water three times for five minutes each, apply 200 μL of NanoSuit Solution Type II (NanoSuit Co., Japan) onto the slide glass. Then, using a spin coater set at 3000 rpm, thinly spread the NanoSuit solution over the tissue. Field emission scanning electron microscopy (SEM) was performed using a Hitachi S-4800 Field Emission Scanning Electron Microscope instrument (Hitachi High-Technologies Corporation, Japan) operated at an acceleration voltage of 5kV. The backscattered electron (BSE) image was taken using a Yttrium-Aluminium-Garnet (YAG)BSE detector (Hitachi High-Tech Corporation, Tokyo, Japan). The immunohistochemically positive region that had been previously observed was identified on the SEM image.

## Discussion

*Listeria sp*. is widely distributed in the environment, including river water and the intestines of animals. Although it is inactivated by heating, it is capable of proliferating even at temperatures below 4°C and in environments with a concentration of up to 12% sodium chloride. Identified sources of infection include ready-to-eat foods that are typically consumed without prior heat treatment, such as dairy products (including natural cheese), processed meats (such as raw ham), seafood products (such as smoked salmon), and prepared foods (such as deli salads and coleslaw) [7,8]. Therefore, it is advised for pregnant women to wash raw vegetables and fruits, store food in the refrigerator, consume products before the expiration date, and adequately heat food before ingestion [7,8]. In the present case, maternal dietary history and preferences obtained via interview revealed no consumption of unpasteurized dairy products or uncooked processed meats. A causative food item could not be identified. Typical symptoms of maternal listeriosis include influenza-like manifestations, such as chills, fever, and myalgia, with the potential for progression to sepsis, meningitis, or central nervous system symptoms [7]. There are also reports of laboratory findings resembling HELLP syndrome, including fever, hemolysis, elevated liver enzymes, and thrombocytopenia. Therefore, febrile nonspecific symptoms during pregnancy should prompt consideration of infectious etiologies [4].

Although this case initially appeared to be a typical intrauterine infection presenting with preterm labor and fetal compromise, certain unique findings were noted. Specifically, the amniotic fluid had a vivid lemon-yellow coloration, and the neonate exhibited highly viscous sputum in the oral cavity. It was speculated that the fetus led to pneumonia and resulting airway secretions, which were the cause of oral obstruction and yellow amniotic fluid. To the best of our knowledge, this color finding has not been previously reported.* Listeria* infection typically results from ingestion of contaminated food and spreads hematogenously across the placenta from the maternal gastrointestinal tract to the fetus; on the other hand, an alternative ascending route from the vagina, via contaminated feces, has also been reported [7]. In the present case, *Listeria monocytogenes* was isolated from neonatal blood, stool, pharyngeal, and otorrhea cultures, as well as from swab cultures of the placental surface. However, maternal vaginal discharge and blood cultures were negative. Placental pathology of *Listeria* infection shows a light yellow microabscess on gross appearance and acute villitis with abscesses on microscopic appearance [9]. This case's histopathological examination of the placenta did not reveal findings typical of transplacental infection, such as intervillositis or villitis (Figures 1D, 3B). Neutrophilic infiltration was predominantly observed in the decidua at the marginal zone of the fetal membrane, near the internal cervical os, which had ruptured during the cesarean section (Figure 1C). Immunohistochemical staining using an anti-*Listeria* antibody showed positivity only in the amniotic epithelium (Figure 3A). Furthermore, scanning electron microscopy with the NanoSuit method clearly revealed numerous* Listeria monocytogenes* organisms in the amniotic epithelium (Figure 4). No neutrophil infiltration or *Listeria* immunostaining positivity was observed in the placental villi (Figures 1D, 3B). These findings strongly suggest that the infection was caused by ascending transmission of *Listeria monocytogenes* from the vagina, following fecal contamination; thus, it is a case of transvaginal infection. However, we have no clear explanation of the negative *Listeria monocytogenes* findings from the vaginal discharge.

A retrospective study of Listeriosis, which used an electronic and manual retrieval system (2008-2017), reported that 89% of maternal cases involved intrauterine *Listeria* infection in mainland China. The total of 514 cases that occurred in the perinatal period consisted of 116 pregnant women and 398 neonates. Among pregnant women, 103 patients had intrauterine infections (89%), in all of which only* Listeria* was isolated from the cervical secretions [10]. The most sensitive diagnostic tests for pregnancy-related Listeriosis are placental swab cultures and neonatal gastric aspirate cultures, both of which demonstrate a sensitivity of 78%. In contrast, a previous study reported that maternal blood cultures were positive for* Listeria* *monocytogenes* in only 33-68% of cases [8]. Severe maternal illness due to *Listeria* is rare, and most cases resolve without antibiotic treatment. Among neonates with pregnancy-associated listeriosis, 62-72% develop bacteremia, 9-13% develop pneumonia, and 13-19% develop meningitis. The neonatal mortality rate ranges from 9-50%, and up to 13% of surviving infants develop neurological sequelae [11]. Regardless of the manifestation of non-reassuring fetal status, when intrauterine infection is suspected during pregnancy, performing maternal bacterial and/or placental swab cultures may improve the diagnostic yield.

## Conclusions

Although it is a rare condition, *Listeria monocytogenes* infection may result in severe consequences for the fetus during pregnancy. In cases of preterm labor associated with intrauterine infection,* Listeria* should be considered as a possible etiology. Detailed interviews about the maternal environment, preferences, and dietary history, along with proactive bacterial cultures from both the mother and neonate, are recommended. Placental histopathological examination may also provide insights into the route of infection.
